# Supplementary material for: AI-assisted discovery of potent FGFR1 inhibitors via virtual screening and in silico analysis
Source: PLoS One. 2025 Sep 11;20(9):e0331837. doi: 10.1371/journal.pone.0331837 (PMC12425237; doi:10.1371/journal.pone.0331837)
Supplement: S1 File — This file contains additional data supporting the findings of this study. (DOCX) [file pone.0331837.s001.docx]

**Supplementary Information**

**AI-assisted discovery of potent FGFR1 inhibitors *via* virtual screening and *in silico* analysis**

Ram Lal (Swagat) Shrestha^1,3,4¶^, Ashika Tamang^1,3¶^, Sandeep Poudel Chhetri^2,3¶^, Nirmal Parajuli^1,3^, Manila Poudel^3^, Shiva M.C.^1,3^, Aakar Shrestha^3^, Timila Shrestha^1,3^, Samjhana Bharati^1,3^, Binita Maharjan^1,3^, Bishnu P. Marasini^3,4,5&*^, Jhashanath Adhikari Subin^3,6&*^

^1^Department of Chemistry, Amrit Campus, Tribhuvan University, Lainchaur, Kathmandu 44600, Nepal

^2^Central Department of Physics, Tribhuvan University, Kirtipur, Kathmandu 44600, Nepal

^3^Kathmandu Valley College, Syuchatar Bridge, Kalanki, Kathmandu 44600, Nepal

^4^Institute of Natural Resources Innovation, Kalimati, Kathmandu 44600, Nepal

^5^Nepal Health Research Council, Ministry of Health and Population, Ramshah Path, Kathmandu 44600, Nepal

^6^Bioinformatics and Cheminformatics Division, Scientific Research and Training Nepal P. Ltd., Bhaktapur 44800, Nepal

^*^For Corresponding authors:

Email address: subinadhikari2018@gmail.com (JAS)

bishnu.marasini@gmail.com (BPM)

^¶^These authors contributed equally to this work.

^&^These authors also contributed equally to this work.

The supplementary information includes 1 table, 13 figures and 1 MDS input file.

**S1 Table:** Binding free energy contributions of key amino acid residues in FGFR1 with top compounds

| **Ligands** | **Residues** | **Average energy ± SD (kcal/mol)** |
| --- | --- | --- |
| **M28** | Leu27 | −0.93±0.51 |
|  | Gly28 | −0.72±0.30 |
|  | Val35 | −1.32±0.33 |
|  | Lys57 | 1.94±1.47 |
|  | Glu74 | 1.45±1.91 |
|  | Ile88 | −0.90±0.29 |
|  | Val104 | −0.66±0.33 |
|  | Glu105 | 1.62±0.62 |
|  | Ala107– | 0.72±0.44 |
|  | Ser108 | 0.98±0.75 |
|  | Lys109 | −0.58±0.30 |
|  | Gly110 | −0.75±0.49 |
|  | Asn111 | −2.38±0.77 |
|  | Glu114 | 1.12±1.02 |
|  | Leu173 | −1.46±0.32 |
|  | Asp184 | 1.23±1.49 |
|  | **LIG** | **−10.12±1.98** |
| **M29** | Gly30 | −1.26±0.46 |
|  | Ala31 | −0.63±0.29 |
|  | Phe32 | −2.30±0.64 |
|  | Gly33 | −1.36±0.50 |
|  | Gln34 | −0.81±0.35 |
|  | Val35 | −1.08±0.29 |
|  | Lys57 | 2.74±2.02 |
|  | Leu59 | −0.55±0.31 |
|  | Asp67 | 0.70±0.97 |
|  | Glu74 | 0.73±1.24 |
|  | Asp166 | 0.99±1.64 |
|  | Arg170 | 1.25±1.18 |
|  | Asp184 | 5.93±4.31 |
|  | Arg198 | 1.24±1.41 |
|  | **LIG** | **−13.57±2.00** |
| **M32** | Leu27 | −1.36±0.55 |
|  | Val35 | −0.94±0.37 |
|  | Lys57 | 0.94±1.19 |
|  | Ala107 | 0.69±0.40 |
|  | Lys109 | −0.61±0.26 |
|  | Leu173 | −1.14±0.29 |
|  | Asp184 | 2.75±1.84 |
|  | **LIG** | **−6.57±1.27** |
| **M34** | Leu27 | −0.73±0.47 |
|  | Gly28 | −0.57±0.41 |
|  | Glu29 | −0.63±0.30 |
|  | Val35 | −1.80±0.36 |
|  | Lys57 | 1.61±1.56 |
|  | Glu74 | −0.82±0.57 |
|  | Val104 | −1.23±0.20 |
|  | Arg170 | 1.11±0.92 |
|  | Asn171 | −1.23±0.46 |
|  | Leu173 | −1.11±0.27 |
|  | Ala183 | −1.73±0.26 |
|  | Asp184 | 3.51±1.40 |
|  | Gly186 | 0.73±0.40 |
|  | Arg189 | −0.58±0.28 |
|  | **LIG** | **−9.87±2.00** |

**
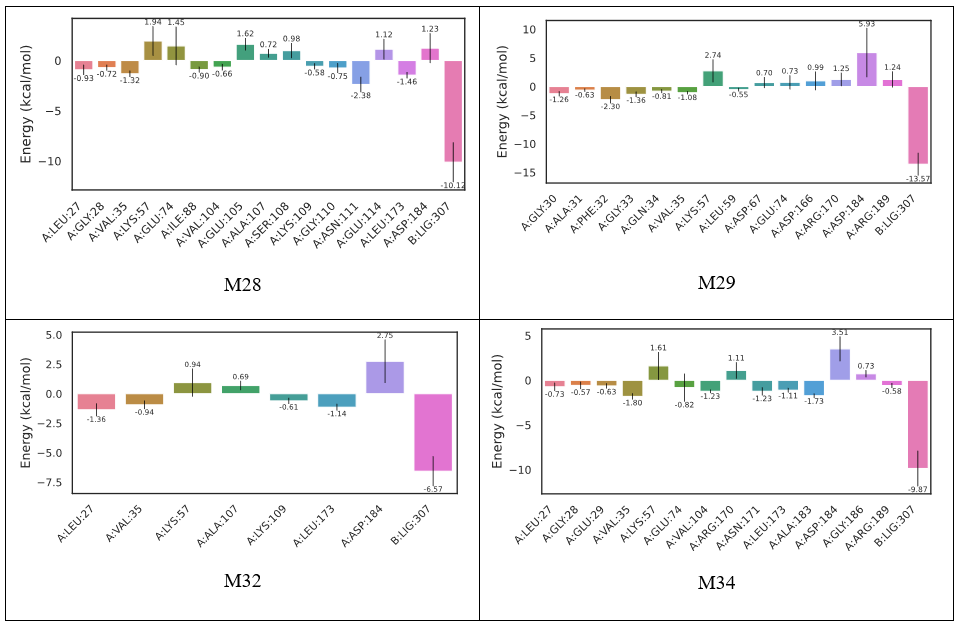
**

**S1 Fig. Bar plots representing per-residue free energy change contributions for top four candidates**

**
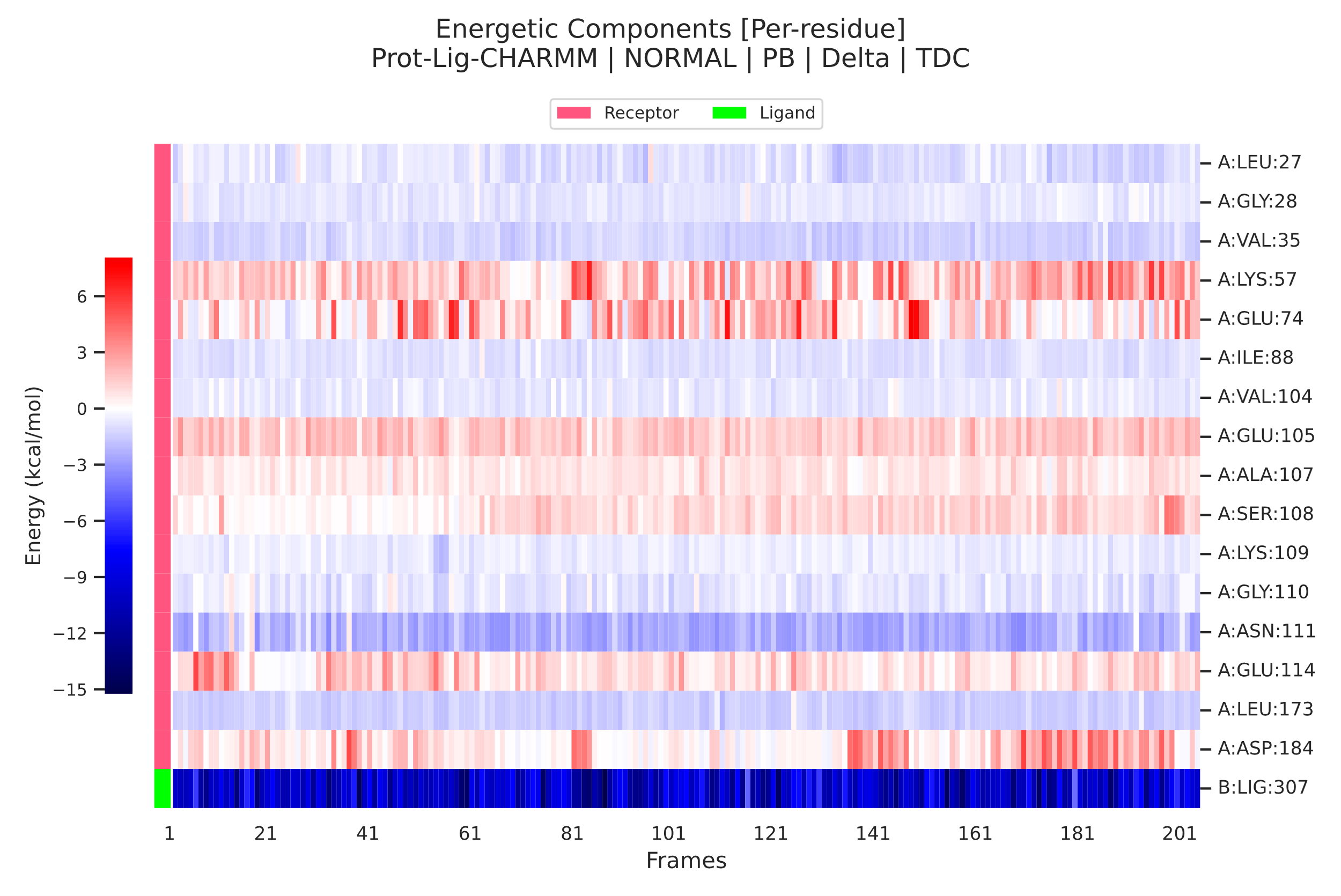
**

**S2 Fig. Heat maps demonstrating the per-residue binding free energy change contributions of active site residues interacting with selected ligands over the final 20 ns of MD simulations for M28-complex**

**
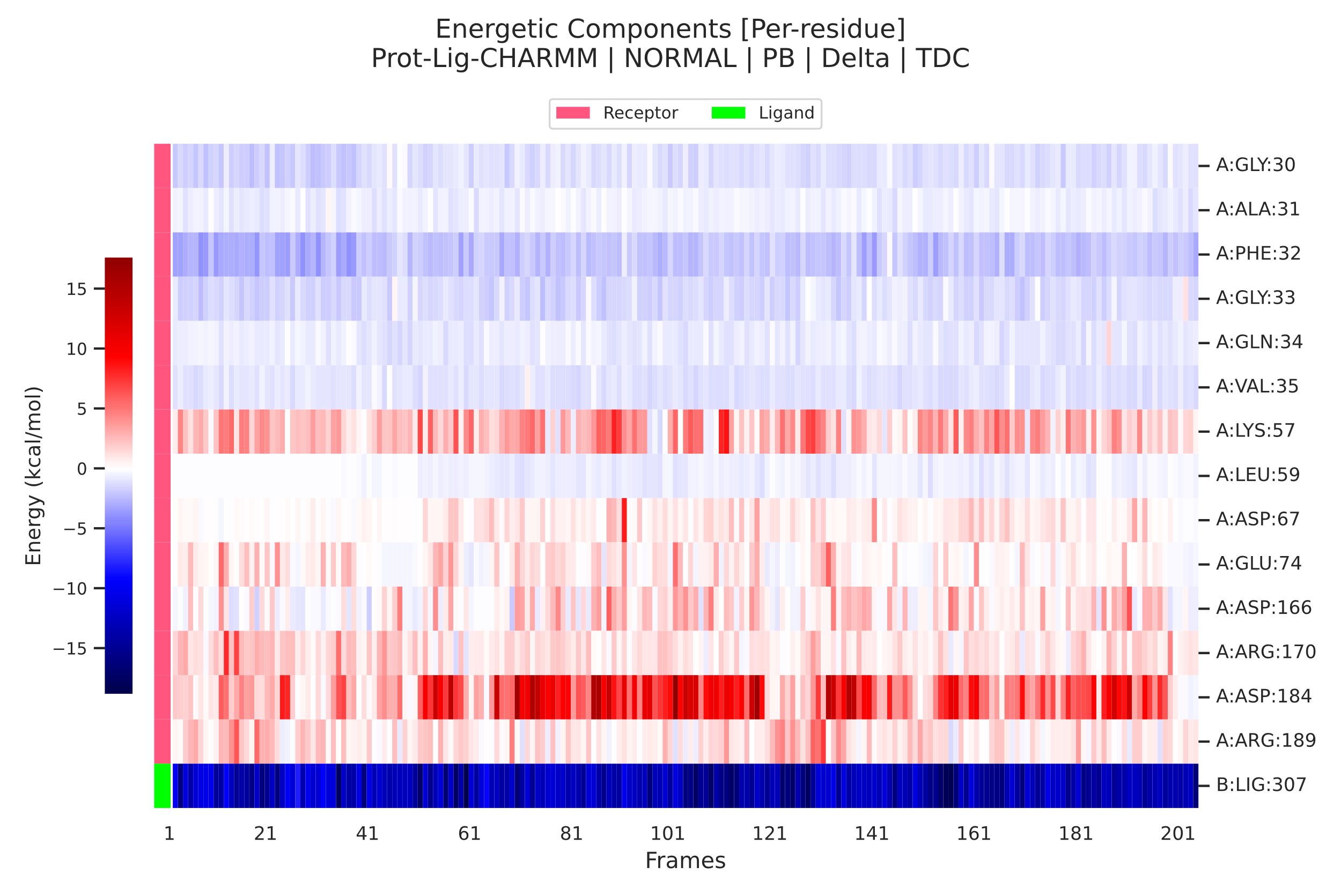
**

**S3 Fig. Heat maps demonstrating the per-residue binding free energy change contributions of active site residues interacting with selected ligands over the final 20 ns of MD simulations for M29-complex
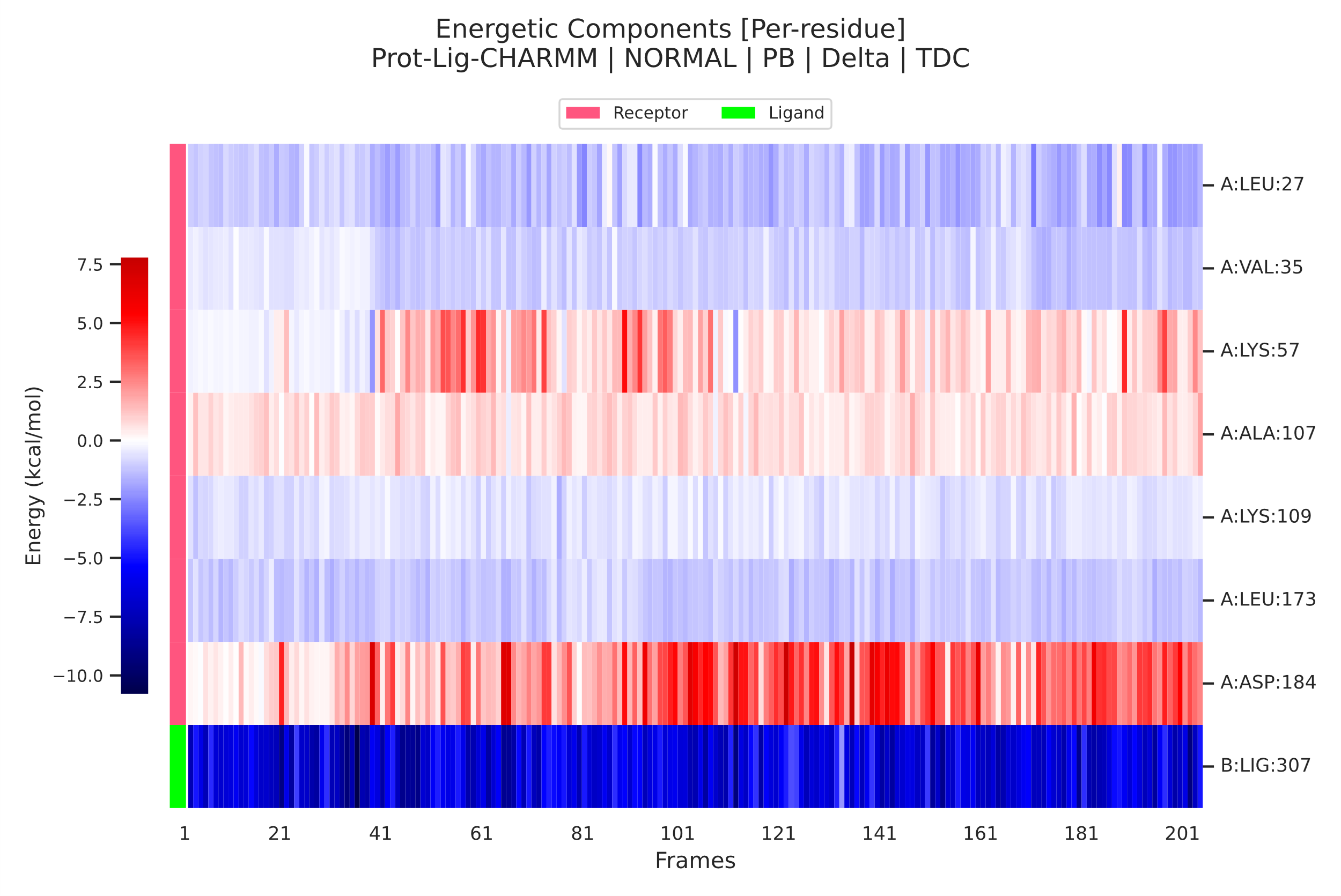
**

**S4 Fig. Heat maps demonstrating the per-residue binding free energy change contributions of active site residues interacting with selected ligands over the final 20 ns of MD simulations for M32-complex**

**
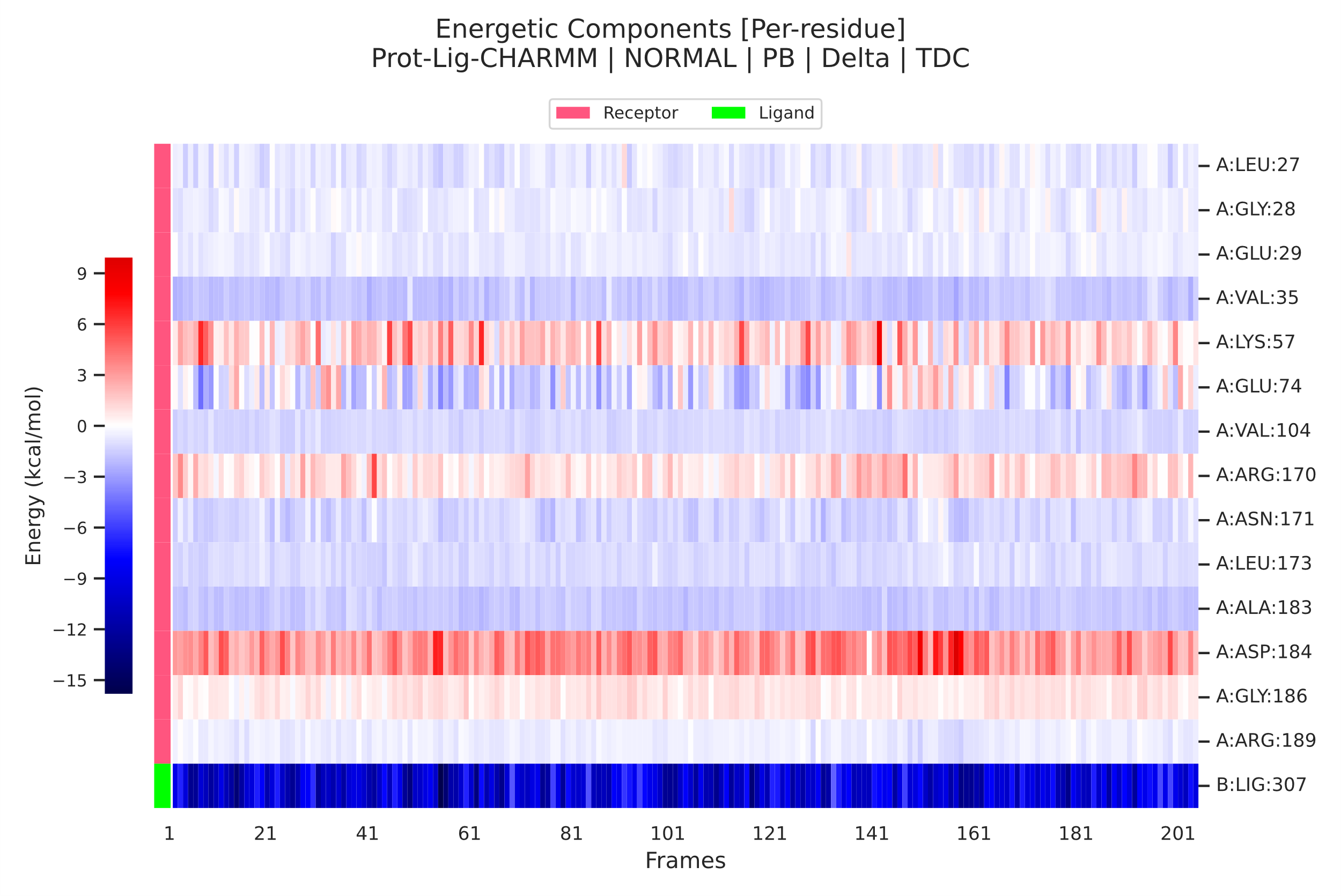
**

**S5 Fig. Heat maps demonstrating the per-residue binding free energy change contributions of active site residues interacting with selected ligands over the final 20 ns of MD simulations for M34-complex**


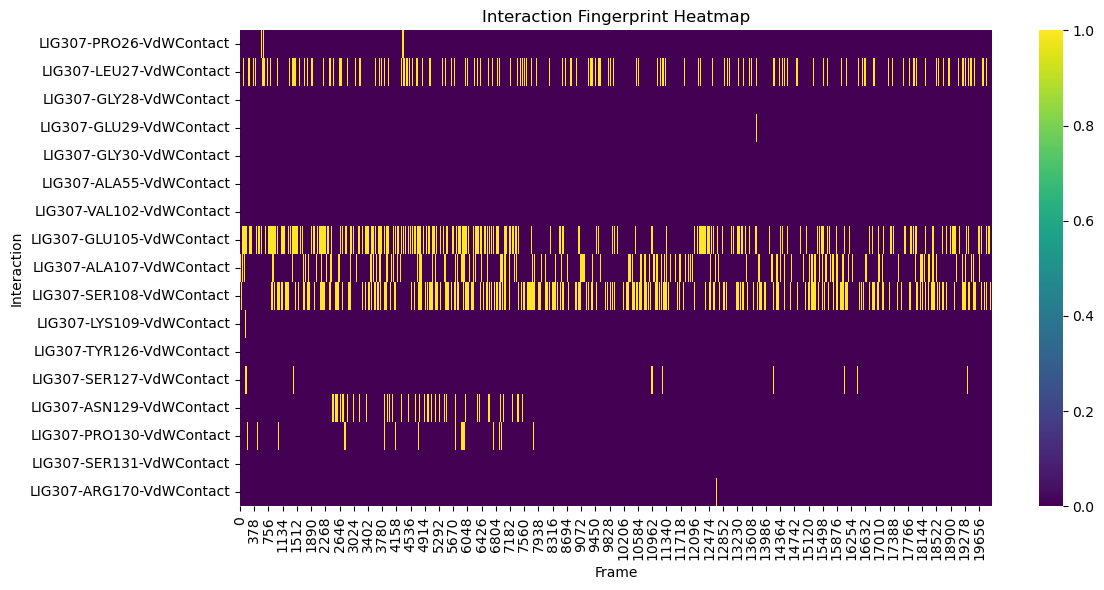


**S6 Fig.** **The interaction fingerprint heatmaps of ligand and amino acid residues of FGFR1 protein during 20,000 frames (200 ns) of MDS of protein-M28 complex**


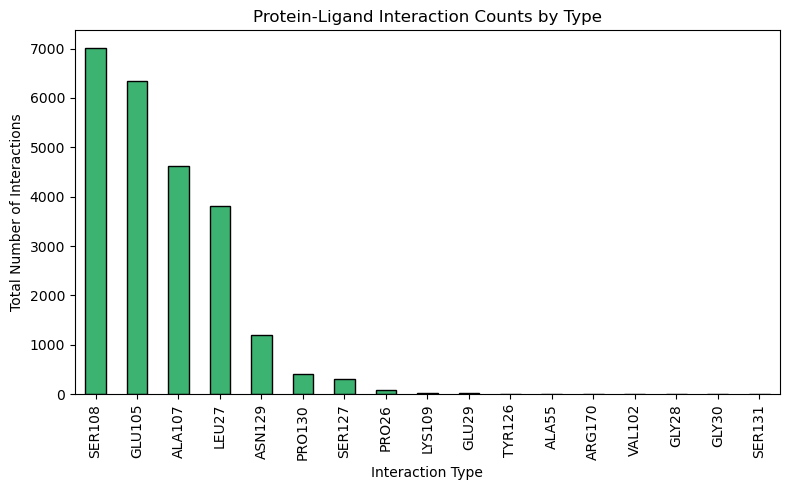


**S7 Fig. Bar plot/histogram demonstrating the amino acid residue interaction count during 20,000 frames (200 ns) MDS in protein-M28 complex**
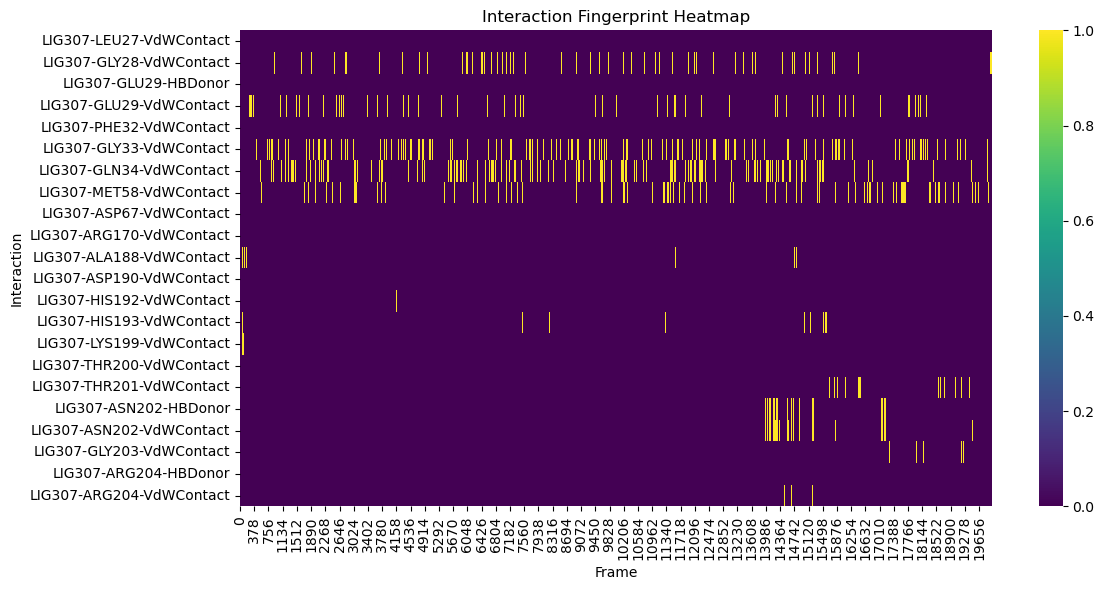


**S8 Fig: The interaction fingerprint heatmaps of ligand and amino acid residues of FGFR1 protein during 20,000 frames of MDS of protein-M29 complex**


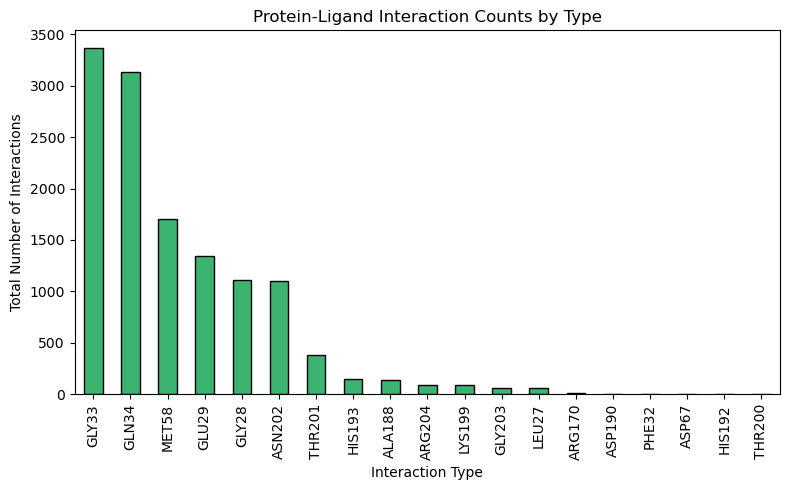


**S9 Fig. Bar plot/histogram demonstrating the amino acid residue interaction count during 20,000 frames (200 ns) MDS in protein-M29 complex**
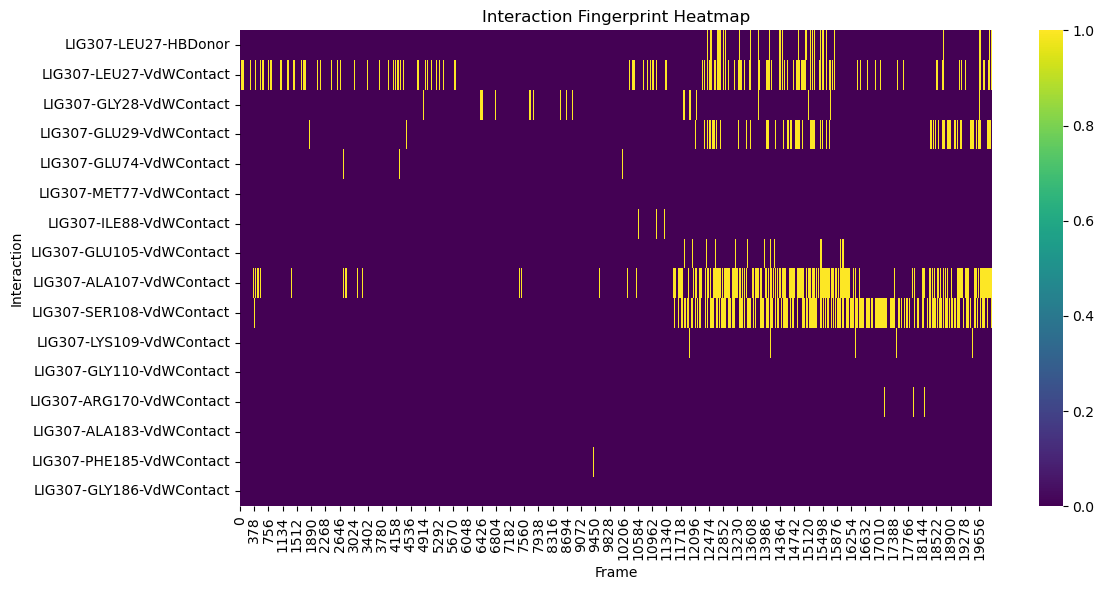


**S10 Fig. The interaction fingerprint heatmaps of ligand and amino acid residues of FGFR1 protein during 20,000 frames (200 ns) of MDS of protein-M32 complex**


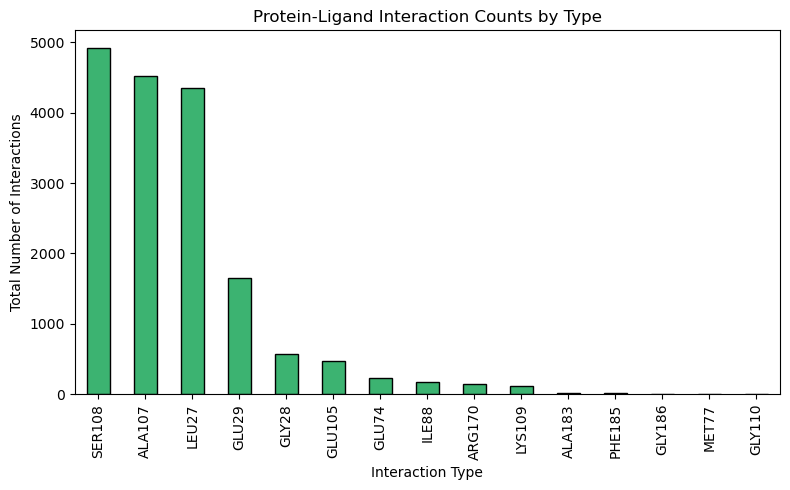


**S11 Fig. Bar plot/histogram demonstrating the amino acid residue interaction count during 20,000 frames (200 ns) MDS in protein-M32 complex**


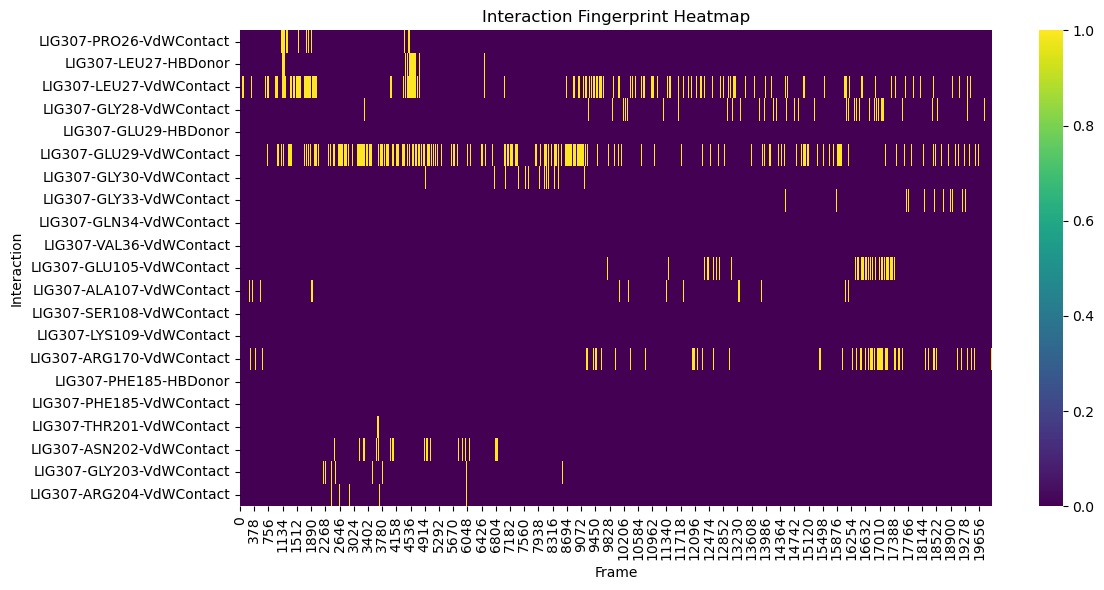


**S12 Fig. The interaction fingerprint heatmaps of ligand and amino acid residues of FGFR1 protein during 20,000 frames (200 ns) of MDS of protein-M34 complex**


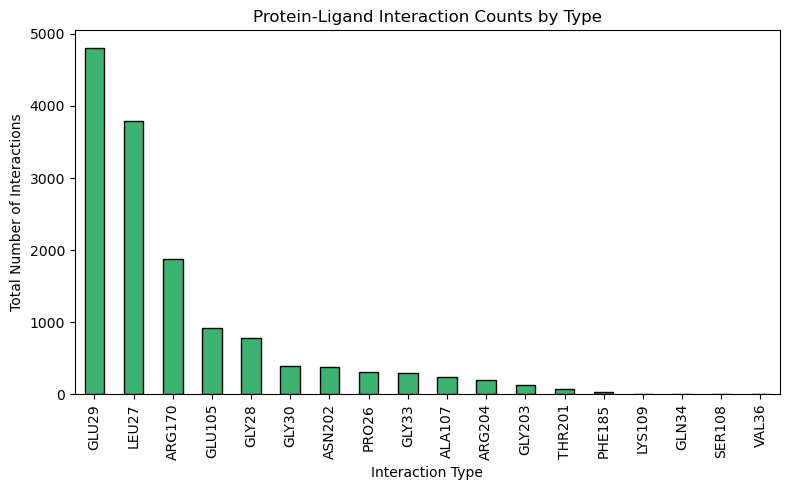


**S13 Fig. Bar plot/histogram demonstrating the amino acid residue interaction count during 200 ns MDS in protein-M34 complex**

**Molecular Dynamics Simulation Input File**

**md.mdp**

title = Protein-ligand complex MDS

; Run parameters

integrator = md ; leap-frog integrator

nsteps = 100000000 ; 2 * 100000000 = 200000 ps (200 nanoseconds)

dt = 0.002 ; 2 femtosecond

; Output control

nstenergy = 5000 ; save energies every ten ps

nstlog = 5000 ; update log file every ten ps

nstxout-compressed = 5000 ; save coordinates every ten ps

; Bond parameters

continuation = yes ; continuing from NPT equilibration

constraint_algorithm = lincs ; holonomic constraints

constraints = h-bonds ; bonds to H are constrained

lincs_iter = 1 ; accuracy of LINCS

lincs_order = 4 ; also related to accuracy

; Neighbor searching and vdW

cutoff-scheme = Verlet

ns_type = grid ; search neighboring grid cells

nstlist = 320 ; largely irrelevant with Verlet

rlist = 1.2

vdwtype = cutoff

vdw-modifier = force-switch

rvdw-switch = 1.0

rvdw = 1.2 ; short-range van der Waals cutoff (in nm)

; Electrostatics

coulombtype = PME ; Particle Mesh Ewald for long-range electrostatics

rcoulomb = 1.2

pme_order = 4 ; cubic interpolation

fourierspacing = 0.16 ; grid spacing for FFT

; Temperature coupling

tcoupl = V-rescale ; modified Berendsen thermostat

tc-grps = Protein_LIG Water_and_ions ; two coupling groups - more accurate

tau_t = 0.1 0.1 ; time constant, in ps

ref_t = 310 310 ; reference temperature, one for each group, in K

; Pressure coupling

pcoupl = Parrinello-Rahman ; pressure coupling is on for NPT

pcoupltype = isotropic ; uniform scaling of box vectors

tau_p = 2.0 ; time constant, in ps

ref_p = 1.0 ; reference pressure, in bar

compressibility = 4.5e-5 ; isothermal compressibility of water, bar^-1

; Periodic boundary conditions

pbc = xyz ; 3-D PBC

; Dispersion correction is not used for proteins with the C36 additive FF

DispCorr = no

; Velocity generation

gen_vel = no ; continuing from NPT equilibration
